# Pilot study on the effects of a 2-week hiking vacation at moderate versus low altitude on plasma parameters of carbohydrate and lipid metabolism in patients with metabolic syndrome

**DOI:** 10.1186/s13104-015-1066-3

**Published:** 2015-03-28

**Authors:** Ivana Gutwenger, Georg Hofer, Anna K Gutwenger, Marco Sandri, Christian J Wiedermann

**Affiliations:** Department of Anesthesiology, Hospital of Bressanone | Brixen, Bressanone | Brixen (BZ), Italy; Department of Anesthesiology, Hospital of Silandro, Schlanders (BZ), Italy; Department of Internal Medicine, Hospital of Silandro | Schlanders, Silandro | Schlanders (BZ), Italy; Data, Methods and Systems Statistical Laboratory, University of Brescia, Brescia (BS), Italy; Department of Internal Medicine, Central Hospital of Bolzano | Bozen, Bolzano | Bozen (BZ), Italy; Interdisciplinary Medical Research Center South Tyrol (IMREST), Bolzano | Bozen (BZ), Italy

**Keywords:** Exercise, Moderate altitude, Hypoxia, Metabolic syndrome, Triglyceride, Cholesterol, Adipokine, Leptin, Adiponectin, Vacation

## Abstract

**Background:**

Hypoxic and hypobaric conditions may augment the beneficial influence of training on cardiovascular risk factors. This pilot study aimed to explore for effects of a two-week hiking vacation at moderate versus low altitude on adipokines and parameters of carbohydrate and lipid metabolism in patients with metabolic syndrome.

**Methods:**

Fourteen subjects (mean age: 55.8 years, range: 39 – 69) with metabolic syndrome participated in a 2-week structured training program (3 hours of guided daily hiking 4 times a week, training intensity at 55-65% of individual maximal heart rate; total training time, 24 hours). Participants were divided for residence and training into two groups, one at moderate altitude (1,900 m; n = 8), and the other at low altitude (300 m; n = 6). Anthropometric, cardiovascular and metabolic parameters were measured before and after the training period.

**Results:**

In study participants, training overall reduced circulating levels of total cholesterol (p = 0.024), low-density lipoprotein cholesterol (p = 0.025) and adiponectin (p < 0.001). In the group training at moderate altitude (n = 8), lowering effects on circulating levels were significant not only for total cholesterol, low-density-lipoprotein cholesterol and adiponectin (all, p < 0.05) but also for triglycerides (p = 0.025) and leptin (p = 0.015), whereas in the low altitude group (n = 6), none of the lipid parameters was significantly changed (each p > 0.05). Hiking-induced relative changes of triglyceride levels were positively associated with reductions in leptin levels (p = 0.006). As compared to 300 m altitude, training at 1,900 m showed borderline significant differences in the pre-post mean reduction rates of triglyceride (p = 0.050) and leptin levels (p = 0.093).

**Conclusions:**

Preliminary data on patients with metabolic syndrome suggest that a 2-week hiking vacation at moderate altitude may be more beneficial for adipokines and parameters of lipid metabolism than training at low altitude. In order to draw firm conclusions regarding better corrections of dyslipidemia and metabolic syndrome by physical exercise under mild hypobaric and hypoxic conditions, a sufficiently powered randomized clinical trial appears warranted.

**Trial registration:**

ClinicalTrials.gov ID NCT02013947 (first received November 6, 2013).

## Background

Risk factors for cardiovascular disease and diabetes mellitus include abdominal adiposity, insulin resistance, impaired glucose tolerance, and arterial hypertension, which are often associated with elevations in fasting triglycerides (TG) and glucose in addition to elevated low-density-lipoprotein cholesterol (LDL-C) and decreased high-density lipoprotein cholesterol (HDL-C) levels [1,2]. This clustering of risk factors is termed the “metabolic syndrome” (MetS) [3]. Increased visceral adipose tissue is integral to the development of MetS, which increases the risk not only of type 2 diabetes and cardiovascular disease complications but also of cancer, sleep disorders, sexual dysfunction, and mortality [4-7].

In individuals with increased adipose tissue, circulating levels of adiponectin, a protein released exclusively from adipocytes that has anti-diabetic and anti-atherosclerotic properties, are decreased [8]. Furthermore, decreased adiponectin is associated with high TG [9] and low HDL-C levels [10,11], as also with hypertension [12], suggesting that adiponectin is involved in the pathogenesis of MetS [13].

Leptin, another adipokine, is also a fat cell-derived mediator that acts centrally on the hypothalamus to reduce food intake and increase energy utilization. In obese persons, concentrations of leptin are high and correlate directly with the mass of white adipose tissue [14]. Through inflammatory and pro-atherogenic properties, also leptin provides a functional link between obesity and cardiovascular disease [15,16].

Exercise training can have profound effects on reducing visceral adiposity and other cardiovascular risk factors associated with MetS [17-19]. Exercise exerts beneficial effects partly through alterations in the adipokine profile, and exercise-induced reductions of leptin may be of particular relevance [20]. It is known that obese persons working at moderate to high altitude lose weight [21-23]. Altitude hypoxia has been identified as an important trigger of this beneficial effect and controlled hypoxia may well become a new therapeutic strategy to improve management of MetS [24,25]. Exercising at moderate altitude may represent a superimposed intermittent hypoxia-like stimulus causing beneficial pre-conditioning effects [26]. Hypoxia-inducible factor pathways are involved in beneficially regulating glucose and lipid metabolism, which might have impact on cardio- and cerebrovascular systems and be responsible for the reduction of cardiovascular mortality at altitude [27,28]. For altitude to exert beneficial effects on lipid parameters, individuals probably do not need to be acclimatized first [29]; however, changes of parameters of glucose metabolism may require prolonged exposure to intermittent hypoxia-like stimuli [30].

In the “Austrian Moderate Altitude Study [31]”, patients with MetS tolerated a 3-week hiking vacation at 1,700 m without any physical problems and showed short-term favorable effects on blood pressure, fat and glucose metabolism [31-34]. To date, however, no study has examined the effect of a hiking vacation at moderate altitude on adipokine levels of patients with MetS [35]. The present pilot study was, therefore, designed to explore the impact of a 2-week hypoxic living and exercise stay on lipid and glucose metabolism as related to circulating levels of adiponectin and leptin. It was hypothesized that moderate exercise under the hypobaric hypoxic conditions at 1,900 meters above sea level would affect lipid metabolism parameters and adipokine plasma levels of patients with MetS more effectively than moderate exercise at 300 meters above sea level.

## Methods

### Study participants

The study was performed on patients with MetS defined according to the 2009 International Diabetes Federation, National Heart, Lung and Blood Institute, American Heart Association, World Heart Federation, International Atherosclerosis Society and International Association for the Study of Obesity criteria by the presence of any three of the following five risk factors [3]: waist circumference, men >102 cm, women >88 cm; fasting TG, >150 mg/dL or drug treatment for elevated TG; serum HDL-C, <40 mg/dL, women <50 mg/dL; arterial blood pressure, >130/85 mmHg or drug treatment for hypertension; fasting plasma glucose, >100 mg/dL or drug treatment for hyperglycemia. The study was approved by the Ethics Committee of the Autonomous Province of Bolzano/Bozen (Italy).

Subjects with MetS were recruited by general practitioners (GPs) between September and October 2013. Forty GPs from South Tyrol (Italy) and from the region of Regensburg (Bavaria, Germany) were invited to collaborate in the recruitment of study subjects. The final participation rate was 20% (8 of 40 GPs invited in writing) who identified 14 subjects with MetS interested and able to participate in the study. Inclusion and exclusion criteria had been checked and approved by the principal investigator who also obtained informed consent upon recruitment. Three to seven days before arrival at the study site, exercise testing on a bicycle ergometer up to maximum physical exhaustion was performed with a stepwise increase of workload at the normal living altitudes of the study subjects.

### Design and outcome

The present study is characterized by a pre-post design type (measurements at baseline T_0_ and at the end of the training period T_1_) [36] with two randomized groups. The aim was to compare, through biomarkers relevant for MetS, subjects’ response under two different experimental conditions: exercise program at 300 m versus 1,900 m above sea level.

The 14 participants were assigned to two groups: one group (n = 8) stayed and exercised under mild hypoxic and hypobaric conditions at 1,900 m altitude for two weeks (Sulden/Solda, Autonomous Province of Bozen/Bolzano, Italy), and the other (n = 6) at 300 m altitude (Nals/Nales, Autonomous Province of Bozen/Bolzano, Italy). Definition of MetS of study subjects is given in Table 1. Altitudes at which participants lived before the study were as follows (mean ± SD): low altitude group: 749.2 ± 157.6 m (range 559–956); moderate altitude group, 785.5 ± 154.4 (range 528–921). There was no adaptation period for individuals at their study altitudes before the start of the exercise intervention. Both groups participated in the supervised training program of hiking three hours a day, four times a week (total training time, 24 hours) at an intensity of 55–65% of the individual maximal heart rate. Heart rate was continuously monitored during the exercise with heart rate monitors Polar FT1 (Polar Electro, Bologna, Italy). Each subject was personally supervised by the exercise therapists during all training sessions in order to be sure that heart rates were maintained within the individually defined target range. During the 2-week intervention period, no specific diet plan was followed, and participants were asked to take their medications as usual. The breakfast was offered as a typical buffet breakfast at the hotel. Lunch and dinner consisted of a starter or soup, main course and dessert each. Drinks were not controlled and included alcoholic beverages.

**Table 1:** **Definition of MetS in study participants**

**Patient no. and code**	**Sex (m/f)**	**Age (yrs)**	**Waist circumference (cm)**	**Fasting TG >150 mg/dL or drug treatment for elevated TG (yes/no)**	**Serum HDL-C (mg/dl)**	**Arterial blood pressure >130/85 mmHg or drug treatment for hypertension (yes/no)**	**Fasting plasma glucose >100 mg/dL or drug treatment for hyperglycemia (yes/no)**
Moderate altitude							
1/003	M	54	125	Yes	38	Yes	Yes
2/006	M	39	122	Yes	31	Yes	Yes
3/007	F	53	136	Yes	56	Yes	Yes
4/008	F	60	132	Yes	39	Yes	Yes
5/010	F	53	77	Yes	44	Yes	No
6/012	M	47	91	Yes	55	Yes	No
7/014	F	56	94	Yes	48	Yes	No
8/015	F	39	97	Yes	62	Yes	No
Low altitude							
9/001	F	66	101	Yes	54	Yes	No
10/002	M	60	115	Yes	44	Yes	Yes
11/004	M	67	113	No	45	Yes	Yes
12/005	F	63	86	Yes	50	Yes	Yes
13/009	F	55	107	Yes	44	Yes	Yes
14/011	M	69	139	Yes	48	Yes	Yes

*Abbreviations*: *M* male, *F* female, *TG* triglycerides, *HDL-C* high density lipoprotein cholesterol.

### Anthropometric, body composition and cardiovascular measurements

On the day of arrival (day 0), baseline examinations (T_0_) were carried out; past medical history was taken and anthropometric data were collected. In the evening of day 0, information was given to participants on how exactly the study will be performed. The next morning (day 1), after an overnight fast, blood samples were collected before training started. Assessments and blood sample collection were repeated on day 14, two days after the last training session on day 12.

Body mass was assessed with a digital scale; body composition as percent body fat was measured using bioelectrical impendance analysis (both, OMRON Karada Scan BF 511). Body mass index (BMI) was determined as the individual’s body mass divided by the square of the height – with the value given in units of kg/m^2^. Waist circumference was measured in centimeters (cm) with a tape ruler between the lower rib margin and the iliac crest, at the end of expiration. Arterial blood pressure was measured (BOSO Medicus Family Automated Device; mmHg) two times in five minutes while the participant was seated; for analysis, the mean of the two measurements was used. Resting heart rate was measured as beats per minute (bpm) while the individual was seated after resting for 5 minutes. The maximal heart rate for each participant was assessed in a standard stress test performed at baseline with a bicycle ergometer. Age- and sex-dependent maximum VO_2_ was deduced from maximum ergometer watts as read from a standardized nomogram.

Blood samples were taken before breakfast at 7:00 a.m., immediately cooled, transported to the nearest hospital laboratory with a maximum transportation time of 45 minutes, centrifuged and frozen. Samples were stored at −80°C until analysis. Thereafter, subjects started their training sessions.

Blood samples taken on day 14 were handled as on day 1, i.e. at 7:00 a.m. before breakfast; cycle ergometer testing and T_1_ examinations of all 14 subjects were both also done on day 14 at the study sites. Exercise testing on a bicycle ergometer up to maximum physical exhaustion was then repeated at the altitude of 300 m in all study subjects.

### Laboratory measurements

Blood samples were collected in heparinized tubes, immediately centrifuged at 4°C and the plasma aliquots obtained were frozen and stored at −80°C for analysis at a later date. Parameters of lipid metabolism, glucose metabolism and inflammation were tested. C-reactive protein (CRP), free fatty acids, total cholesterol (TC), TG, and HDL-C measurements were performed on the automated clinical chemistry analyzer (ADVIA 1800; Siemens Healthcare Diagnostics, Eschborn, Germany). The inter-assay coefficients of variation were 0.9, 1.6, 1.4, 2.0 and 0.9%, respectively. Immunoassay systems (ADVIA Centaur) were used to measure plasma levels of insulin and C-peptide with inter-assay coefficients of variation of 2.8 and 3.2%, respectively. Immunoenzyme analysis (sandwich ELISA) was used to measure concentrations of total plasma leptin (DGR, Siemens BEP) and plasma adiponectin (BioVendor, Siemens BEP). The inter-assay coefficients of variation were 11.5 and 7.0% for leptin and adiponectin, respectively. Plasma glucose concentrations were measured using a chemistry-Immuno analyzer (Olympus AU2700™); the inter-assay coefficient of variation was 1.8%.

### Sample size and statistical analysis

Sample size was calculated considering a repeated measure ANOVA, setting the significance level to α=5%, the power to β=80%, the correlation among repeated measures to ρ=0.75 and setting to 0.08 (moderate effect size) the minimum detectable effect sizes (partial eta-squared) for the within factor (pre/post training) and the within-between interaction (altitude × training). The required total sample size is 7 subjects per group.

Two-way repeated measures ANOVA (with main effects of altitude and training, and altitude × training interaction) was performed, followed by pairwise comparisons when one of the ANOVA terms was statistically significant (p < 0.05). The statistical test used to compare pre and post measures was the t-test for paired samples. The pre/post relative change for TG and leptin measurements was calculated as follows:

$$ \Delta \mathrm{X}\%=100\frac{{\mathrm{X}}_1\hbox{-} {\mathrm{X}}_0}{{\mathrm{X}}_0}, $$

where X_0_ and X_1_ were the values of the outcome X measured at baseline and at the end of the training program. Association between TG and leptin pre-post relative changes was estimated using the Spearman’s rank correlation coefficient.

Effects were considered statistically significant at p < 0.05 and borderline significant for p between 0.05 and 0.10. Statistical analyses were performed using R (version 3.1.1; R Foundation for Statistical Computing, Vienna, Austria) statistical software.

## Results

All 14 of the study subjects (mean age: 55.8 years, range: 39–69 years, 6 males and 8 females) diagnosed with MetS who participated in the physical training protocol completed the program. Study participants assigned to the 1,900 m altitude group (n = 8) were 3 men and 5 women aged 50.1 ± 7.8 years (mean ± standard deviation, range 39–60), those of the 300 m altitude group (n = 6) were 3 men and 3 women aged 63.3 ± 5.2 years (range 55–69).

Quantitative data on the training are given in Table 2. Data on the anthropometric, cardiovascular, and the biochemical profile of the subjects before and after the cardiovascular physical training at low or moderate altitude are given in Tables 3 and 4. Effect sizes and statistical significance of main effects and interaction effect for altitude and training are also reported.

**Table 2:** **Characteristics of the 2-week outdoor hiking intervention at low and moderate altitudes**

**Characteristics**	**Low altitude**	**High altitude**
**Walking distance (meter)**	39,500	39,000
**Exercise duration (min)**	1,440	1,440
**Walking speed (km/h)**	1.65	1.62
**Walking elevation gain (meter)**	2.030	2.054

**Table 3:** **Mean values for anthropometric and physiological variables, before (T**_**0**_ **) and after (T**_**1**_ **) the training program in the low (N=6) and moderate (N=8) altitude study groups**

**Variable**	**Low altitude**		**Moderate altitude**		**Altitude**		**Training**		**Altitude x Training**	
	**T**_**0**_	**T**_**1**_	**T**_**0**_	**T**_**1**_	**η** ^**2**^_***P***_	**P**	**η** ^**2**^_***P***_	**P**	**η** ^**2**^_***P***_	**P**
**Body mass (kg)**	88.6 ± 20.2	89.3 ± 18.4	91.8 ± 22.5	91.2 ± 22.7	0.004	0.829	0.000	0.966	0.130	0.206
**BMI (kg/m2)**	32.3 ± 4.2	32.6 ± 3.4	31.1 ± 5.3	30.9 ± 5.4	0.024	0.594	0.005	0.806	0.157	0.469
**WC (cm)**	110.2 ± 17.6	108.7 ± 16	109.2 ± 22	104.0 ± 18.4	0.013	0.701	0.159	0.158	0.077	0.338
**Body fat (%)**	37.8 ± 6.4	36.5 ± 8.2	37.5 ± 7.5	36.9 ± 7.0	0.000	0.990	0.158	0.160	0.021	0.621
**Visceral fat (%)**	16.0 ± 6.4	16.0 ± 6.3	12.2 ± 4.9	12.0 ± 5.1	0.120	0.226	0.058	0.408	0.058	0.408
**Muscle mass (%)**	26.7 ± 3.2	28.6 ± 5.3	27.5 ± 3.7	27.9 ± 3.6	0.000	0.974	0.190	0.119	0.100	0.271
**Syst. BP (mmHg)**	158.2 ± 21.8	143.7 ± 18.1	134.2 ± 20.9	138.1 ± 10.2	0.180	0.131	0.162	0.153	0.367	0.022
**Diast. BP (mmHg)**	88.7 ± 10.0	85.5 ± 8.1	85.9 ± 9.6	86.9 ± 6.8	0.002	0.865	0.019	0.640	0.066	0.375
**Max. syst. BP (mmHg)**	212.3 ± 25.9	187.5 ± 14.7	181.8 ± 33.4	184.3 ± 24.4	0.118	0.250	0.340	0.036	0.397	0.021
**Resting HR (bpm)**	68.2 ± 6.8	65.3 ± 7.9	75.8 ± 11.7	71.2 ± 10.9	0.148	0.174	0.146	0.177	0.009	0.750
**Max. HR (bpm)**	143.0 ± 15.2	144.7 ± 20.9	154.1 ± 22.6	148.9 ± 22.9	0.041	0.508	0.033	0.550	0.113	0.261
**Age-adjust. max. CF (%)**	92.2 ± 11.1	95.3 ± 14.2	90.3 ± 11.6	91.0 ± 9.7	0.021	0.633	0.140	0.207	0.061	0.415
**Power (W=J/s)**	133.3 ± 25.8	138.3 ± 36.0	189.3 ± 82.7	197.1 ± 102.6	0.162	0.173	0.064	0.404	0.003	0.851
**VO**_**2**_ **max (ml/min)**	1754 ± 383.8	1816 ± 513.9	2335 ± 1058.4	2417 ± 1231.7	0.106	0.255	0.079	0.331	0.002	0.887

Effect sizes from a 2-way repeated measures ANOVA: main effect of altitude, main effect of training and altitude-training interaction.

Data are mean ± standard deviation; η^2^_*P*_ = effect size (partial eta squared; ‘small’ effect size, 0.01 < η^2^_*P*_ ≤ 0.06; ‘medium’ effect size, 0.06 < η^2^_*P*_ ≤ 0.14; ‘large’ effect size, η^2^_*P*_ > 0.14); P = significance level of the F statistic; N = number; T = time point; BMI = body mass index; WC = waist circumference; Max. syst. BP = maximum systolic blood pressure; Diast. BP = diastolic blood pressure; HR = heart rate; bpm = beats per minute; Max. = maximum; Age-adjust. = age-adjusted; CF = cardiac frequency.

**Table 4:** **Mean values for blood lipid parameters before (T**_**0**_ **) and after (T**_**1**_ **) the training program in the low (N=6) and moderate (N=8) altitude study groups**

**Variable**	**Low altitude**		**Moderate altitude**		**Altitude**		**Training**		**Altitude × training**	
	**T**_**0**_	**T**_**1**_	**T**_**0**_	**T**_**1**_	**η** ^**2**^_***P***_	**P**	**η** ^**2**^_***P***_	**P**	**η** ^**2**^_***P***_	**P**
**Total cholesterol (mg/dl)**	195.5 ± 29.4	188.8 ± 20.2	206.6 ± 36	179.9 ± 40.4	0.000	0.950	0.357	0.024	0.167	0.147
**HDL-C (mg/dl)**	47.5 ± 4.0	47.8 ± 7.7	46.6 ± 10.6	47.0 ± 11.6	0.002	0.866	0.011	0.716	0.000	0.983
**LDL-C (mg/dl)**	145.2 ± 30.8	131.7 ± 23.6	145.6 ± 33.3	120.2 ± 37.4	0.010	0.734	0.353	0.025	0.048	0.450
**TG (mg/dl)**	117.5 ± 48.5	121.5 ± 31.0	186.4 ± 93.1	126.8 ± 58.8	0.101	0.269	0.233	0.080	0.284	0.050
**Free fatty acids (mg/dl)**	13.6 ± 5.9	12.8 ± 8.3	11.5 ± 5.5	13.6 ± 4.8	0.003	0.846	0.025	0.590	0.106	0.257
**Adiponectin (mg/l)**	10.1 ± 2.8	9.1 ± 3.1	9.5 ± 2.9	8.1 ± 2.0	0.025	0.591	0.642	<0.001	0.027	0.578
**Leptin (μg/l)**	11.2 ± 5.3	11.8 ± 5.6	11.2 ± 9.1	8.2 ± 7.3	0.018	0.649	0.108	0.251	0.217	0.093
**Insulin (U/l)**	16.8 ± 10.2	18.3 ± 9.8	14.7 ± 5.8	15.7 ± 8.0	0.026	0.579	0.041	0.486	0.003	0.859
**Glucose (mg/dl)**	107.3 ± 23.5	105.8 ± 16.9	104.9 ± 18.1	103.3 ± 20.3	0.005	0.809	0.020	0.628	0.000	0.985
**C-peptide (U/l)**	3.6 ± 1.2	3.5 ± 1.0	3.5 ± 1.1	3.3 ± 1.3	0.005	0.818	0.045	0.467	0.006	0.786
**C-reactive protein (mg/dl)**	0.20 ± 0.14	0.21 ± 0.11	0.43 ± 0.40	0.35 ± 0.36	0.128	0.209	0.021	0.652	0.026	0.580

Effect sizes from a 2-way repeated measures ANOVA: main effect of altitude, main effect of training and altitude-training interaction.

Data are mean ± standard deviation; η^2^_*P*_ = effect size (partial eta squared; ‘small’ effect size, 0.01 <η^2^_*P*_ ≤ 0.06; ‘medium’ effect size, 0.06 < η^2^_*P*_ ≤ 0.14; ‘large’ effect size, η^2^_*P*_ > 0.14); P = significance level of the F statistic; N = number; T = time point; BMI = body mass index; WC = waist circumference; Max. syst. BP = maximum systolic blood pressure; Diast. BP = diastolic blood pressure; HR = heart rate; bpm = beats per minute; Max. = maximum; Age-adjust. = age-adjusted; CF = cardiac frequency.

Significant altitude × training interactions with large effect size were seen for systolic blood pressure at rest and maximum systolic blood pressure in the cycle ergometer test (p = 0.022 and 0.021, respectively; Table 3). Pre-post pairwise comparisons revealed that mean systolic blood pressure at rest and maximum systolic blood pressure in ergometry were reduced by training at low altitude (p = 0.040 and p = 0.036, respectively) but not at moderate altitude (p = 0.424 and 0.792, respectively).

Borderline significant altitude x training interactions with large effect sizes were noted for TG and leptin (p = 0.050 and p = 0.093, respectively; Table 4). Pre-post comparisons of these parameters showed significant reductions at moderate (p = 0.025 and 0.015, respectively) but not at low altitude (p = 0.838 and 0.765, respectively).

Large and statistically significant main effects of training were observed for levels of total cholesterol, LDL-C and adiponectin (p = 0.024, p = 0.025 and p < 0.001, respectively; Table 4). Training significantly reduced adiponectin levels both at moderate (p = 0.005) and low altitude (p = 0.044). Total cholesterol and LDL-C showed significant pre-post reductions of mean levels only for moderate altitude (p < 0.001).

Using repeated measures mixed models (also known as hierarchical random effects models), we re-evaluated the differences in the distribution of the study parameters between the two altitude groups, correcting for the VO_2_max baseline values. Each model has the following X covariates: altitude group (low vs moderate), time point (pre vs. post training), “altitude × time” interaction and VO_2_max baseline values. These analyses largely confirmed the previous results.

**Figure 1:**
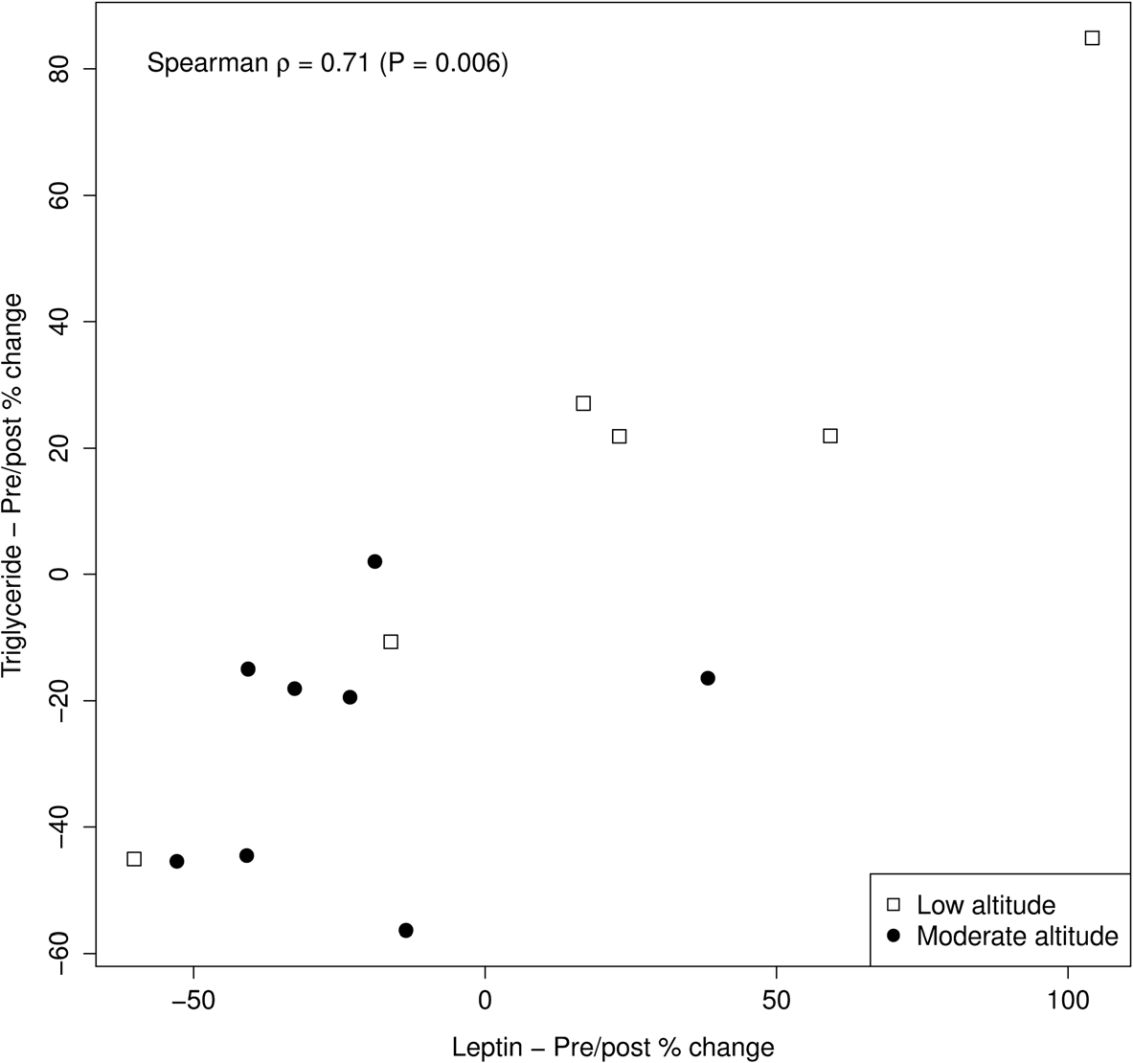
**Pre- and post-training change (percentage) in leptin and triglyceride levels.**

Relative changes of triglyceride and leptin levels showed a statistically significant correlation (Spearman ρ = 0.71, p = 0.006, Figure 1).

## Discussion

The main new finding of the current study is that a 2-week hiking vacation at moderate altitude was more effective in acutely reducing elevated plasma leptin levels of patients with MetS than a comparable hiking vacation at low altitude, and that this change is correlated with reductions of triglyceride levels. The study additionally suggests that a short-term training intervention in patients with MetS reduces total cholesterol and LDL-C levels even in the absence any dietary restrictions.

It is worth pointing out that these findings have to be interpreted with caution due to the preliminary nature and some intrinsic limitations of the present study. Firstly, the limited sample size allows to detect only moderate and large effects for the within (training) effect and the within-between (training × altitude) interaction. Secondly, a moderate imbalance is present in the assigned number of subjects in the low vs. moderate altitude groups (n = 6 and 8, respectively). Thirdly, due to the small sample size, randomization did not achieve complete balance at baseline for the distribution of gender and age in the low vs. moderate altitude groups; however, the imbalance of physiological and blood lipid parameters at baseline in the two altitude groups is not a problem. The pre/post design of the study and the repeated measures analysis of variance allowed efficient removal of the effect of imbalance at T_0_.

The present pilot study has some interesting results on the effects of training at different altitudes on the carbohydrate and lipid parameters in MetS. For firm conclusions, a future randomized controlled trial with an adequate sample size is needed.

Low intense physical exercise in normobaric hypoxia leads to more weight loss in obese people than low intense physical exercise in normobaric sham hypoxia [37]. In patients with MetS, physical training under hypoxic and hypobaric conditions of moderate altitude beneficially affects not only metabolic parameters but also anthropomorphic and hemodynamic parameters [31-34]. In the current study, anthropomorphic and body composition parameters did not significantly change in either study group (Table 2). Most likely this is due to the small sample size, but may also be related to the short intervention time of 2 weeks as well as the fact that training intensity was designed to be moderate and suitable for untrained people with metabolic and cardiovascular risk factors. Improved cardiorespiratory fitness has been observed in a study with similar design and comparable training intensity; however, the duration of intervention was 3 weeks as compared to 2 weeks of the present study [31,32]. Moreover, as no nutritionally standardized menus were offered, it may well be that caloric intake may have been increased during the hiking vacation period thus reducing the effects of training on body weight and composition.

Dose–response relationships between exercise training volume and blood lipid changes have been intensively investigated in the past. Data suggest that exercise can beneficially alter blood lipids at low training volumes, and HDL-C and TG are more amenable to exercise than others with greater exercise training duration and caloric expenditure associated with more marked improvements [37,38]. Favorable changes of HDL-C and TG levels by moderate endurance training (70% VO_2_max, 30 min/day, 3–4 days/week) of up to eight weeks duration have been observed beyond a duration of three weeks [39]. In healthy adults, three weeks of daily running of 6.5 km at 19 km/h (high intensity) or 3.2 km at 10 km/h (low intensity) increased HDL-C significantly by 10% in both groups and reduced TG by 37% and 28% in the high and low intensity group, respectively [40]. In a tree-week cycle ergometry study in 12 healthy males exercising daily at 58% of their maximal oxygen uptake for a mean of 116 min/day in order to induce a daily energy deficit of 1000 kcal, plasma TG decreased and HDL-C concentration increased significantly [41]. Whether these improvements in lipid metabolism are the sole consequences of the short exercise programs remains questionable.

In the Austrian Moderate Altitude Study [31], the effects of a 3-week exercise training program were investigated at 1,700 m altitude in subjects with metabolic syndrome that included 12 moderate-intensity guided hiking tours 4 times/week at 55-65% heart rate maximum with a total exercise time of 29 h; HDL-C levels increased significantly during the training program by day 19, whereas no changes were seen for TC and LDL-C levels; HDL-C levels returned to baseline levels 7 to 10 days after the training program [31,32]. An explanation for this observation may be that endurance exercise acutely lowered TG levels and increases HDL-C, a phenomenon known to occur for up to 48 h after acute exercise [42,43]. In our pilot study, the duration of comparable exercise intensity was shorter (two weeks) and blood samples were drawn 48 hours after the last training session when acute effects of exercise training probably have diminished but may not yet have entirely disappeared.

Exercise training and a single session of exercise exert distinct and interactive effects on lipids and lipoproteins [44]. Single sessions of exercise transiently reduce serum TG which occurs after a delay of hours, is generally maximal on the day following exercise [44-47] and usually disappears until day 3 [43]. The effect can be blunted when the energy and carbohydrate deficit induced by exercise is replaced during the hours post-exercise [48,49]. In the current pilot study, effects of the two-week exercise training program on fasting TG levels were assessed 48 hours after the last training session, when acute effects of the last training bout may not yet have entirely disappeared. Even though exercise-induced energy and carbohydrate deficit may have been sufficiently replaced in our study because dietary intake was not controlled, this remains as a possible confounding factor. Results, therefore, have to be interpreted with caution regarding acute vs. chronic exercise effects on lipid metabolism parameters.

Decreased leptin levels in response to exercise training are typically associated with reductions in body mass, BMI and body fat mass [50-53]. In one study of 9 middle-aged sedentary women without dietary restrictions, however, walking and/or running for 45 min 4 times/week at 85% heart rate maximum for a total of 12 weeks significantly reduced leptin levels by more than 17%, whereas body mass, body mass index and percent body fat mass were not significantly changed [54]. Thus, our finding of significantly reduced leptin levels by a 2-week training intervention in the absence of significant changes in body weight and composition is not contradictory.

Altitude training can beneficially affect the metabolic system, and has the potential to serve as a non-pharmacological or recreational intervention regimen for correcting MetS [55]. In our study, fasting plasma glucose, insulin and C-peptide levels were not significantly affected by the 2-week hiking vacation. This observation is compatible with previous findings showing that short-term moderate altitude hypoxia living and physical endurance training of 3 weeks or less can improve results of glucose tolerance testing but does not affect fasting glucose levels [56,57].

Effects of hypoxic training on parameters of lipid metabolism studied so far have been mixed. While improvements in total cholesterol (−4.2% to −30%) and LDL-C (−2.6% to −14.3%) have been reported as a result of hypoxic training, available evidence does not substantiate hypoxic training for the improvement of HDL-C and TG (for review, see [55]). In the study subjects with MetS analyzed here, total cholesterol and low-density-lipoprotein cholesterol significantly improved upon 2 weeks of moderate endurance training (both, large effect size) with no significant changes in HDL-C levels. The observed decrease in triglyceride levels showed a borderline statistical significance that may be related to imbalance of baseline levels between the two study groups (Table 3). Significant findings including the correlation of triglyceride changes with leptin changes (Figure 1) confirm the effectiveness of hypoxic training on the modulation of dyslipidemia in MetS.

Increased triglyceride levels independently contribute to cardiovascular risk [58]. This study’s finding of reductions not only of total and low-density lipoprotein cholesterol but of potentially stronger reduction of triglyceride levels by training at moderate altitude is therefore of potential relevance to efforts at reducing cardiovascular risk. However, as mentioned before, the small sample size of the current study and baseline imbalances in triglyceride levels between the moderate and low altitude training groups may confound conclusions. In order to confirm the effectiveness of hypoxic training on the modulation of metabolic cardiovascular risk factors, plasma levels of leptin were therefore further assessed.

Mechanisms by which exercise training improves plasma HDL-C and TG are not entirely understood. Favorable effects of exercise on lipoprotein metabolism may be mediated by favorable changes in body weight and composition, as well as by enhancements in hepatic insulin sensitivity and blood flow, and increase in peripheral lipoprotein lipase (LPL) activity [59]. Exercise training increases hepatic lipases and LPL activity in tissue and muscle. It is suggested that LPL and lower very-low-density lipoprotein (VLDL) and chylomicron TG levels are involved. These changes enhance reverse cholesterol transport and contribute to HDL maturity; TGs are exchanged for cholesterol esters in HDL and LDL and are hydrolyzed by lipases [60]. Due to the low volume and intensity of exercise training in our pilot trial and uncontrolled dietary intake, significant changes in physical fitness, body weight and composition as well as parameters of glucose metabolism have not been observed, however, observed changes in blood lipids need to be confirmed in future studies.

Whereas the effect of exercise on adiponectin levels in healthy and obese subjects was conflicting in the available literature, increased plasma levels of leptin in obese, prediabetic, or MetS patients were consistently reduced by exercise, a finding largely independent of type and duration of exercise protocols tested [20].

Adipokine responses to exercise vary depending on the exercise intensity levels [61,62]. Acute and short-term bouts of exercise do not affect leptin levels in healthy individuals [63] but can reduce leptin levels in diabetic patients [52] who seem to be more responsive to the leptin lowering effects of exercise. Intensity and duration of exercise tested in the current study was defined by patient characteristics including sedentary life style and MetS, and the vacational setting, i.e. two weeks duration, respectively. In our study, results suggest that duration and intensity of physical exercise training tested may be sufficient because plasma leptin levels were significantly reduced in the moderate-altitude but not in the low-altitude group. As the percentage of reduction in plasma leptin levels was closely related to the percentage of reduction in triglyceride levels (Figure 1), these observations corroborate the observed greater effectiveness of short-term training at moderate altitude as compared to low altitude on cardiovascular risk parameters of lipid metabolism in MetS. Leptin plasma levels were significantly reduced in daughters of patients with type 2 diabetes, who, in a 7-week training program, exercised a total of 1,450 min at 74% of their maximum heart rate [64]. In our study, plasma leptin levels also fell in patients with MetS who completed a total of 1,440 min of exercise in a 2-week training program at a maximum heart rate between 55 and 65%. This suggests that potentially beneficial adipokine changes may be achieved in a concentrated 2-week training program even at a somewhat lower maximum heart rate than in previous studies when performed under hypoxic conditions.

Circulating leptin levels correlate with body mass index, blood pressure, total cholesterol, triglyceride, and inflammatory markers; these correlations persist also after body mass index adjustment [65]. The mechanisms involved in the effects of exercise training under hypoxic and hypobaric conditions on plasma leptin and triglyceride levels that strongly correlate with each other remain speculative. Factors potentially contributing to the observed reduction of leptin levels by exercise training may include the effect of hypoxia itself, as serum leptin level decreases when altitude increases, and this association helped to explain the lower cardiovascular mortality rate at high altitude [66]. Training at hypoxic conditions may increase the basal metabolic rate as the result of improved substrate utilization and mitochondrial oxidative capacity via signaling pathways that stimulate GLUT-4 transport [67]. In addition, epinephrine levels increased as a result of hypoxic training [68], which may give rise to increased glycolysis. Training at hypoxic conditions allows a higher relative intensity to be achieved, which would reduce the mechanical strain of higher workloads while gaining similar benefits [69].

Serum leptin levels are correlated positively with insulin resistance, independent of body weight or adiposity, both in normoglycemic and in diabetic patients [70,71]. Since exercise training beneficially affects insulin resistance, mechanisms involved in leptin responses might be related. Many factors regulate leptin expression and secretion from adipocytes, including nutrients, steroid and thyroid hormones, and cytokines [72]. Adipose tissue is richly innervated by sympathetic fibers driving lipolysis during fasting, cold exposure and exercise; beta-adrenergic stimulation decreases leptin release from fat cells [73]. Muscle is a direct and indirect target of adipokines that regulate metabolism, and conversely, releases myokines like irisin, a hormone produced by skeletal muscle in response to exercise that can affect adipose function by various overlapping mechanisms including sympathetic nervous system-induced signaling specifically to brown adipose tissues [73-75]. Beyond local interaction between muscle and adipocytes by adipokines and myokines, circulating irisin predicts insulin resistance onset in association with weight regain and reflects body fat mass, suggesting that the irisin circulating levels are conditioned by adiposity level [76]. If exercise-induced irisin might exert direct effects on leptin production and release from adipocytes is currently not known [77]. Irisin levels have not been assessed in this pilot study but should be further investigated in the particular setting of non-pharmacologic exercise interventions in patients with MetS as it might be involved on its biological mechanism.

Carbohydrate and lipid metabolism are greatly influenced by diet. Observed differences between treatments at low and moderate altitude could be attributed to variation in food intake. Since the pilot study was intended to investigate metabolic effects of hiking during a 2-week vacation, the design was as pragmatic as possible. Therefore, daily dietary intake was not controlled. This is a limitation of the study and precludes mechanistic interpretations. It needs to be underlined that in studies investigating fat metabolism adaptions in response to exercise, as such diet control is of paramount importance and should become part of the actual study protocol.

Lack of documented quantitative data of training of the two intervention groups is another limitation.

Hiking vacations at moderate altitude may become an interesting non-pharmacological intervention in patients with MetS, as such an intervention of 2-to-3 weeks duration not only improved perceived health in individuals with MetS [78] but also cardiovascular risk factors.

## Conclusions

Results of this small pilot study suggest that in patients with metabolic syndrome, a 2-week hiking vacation at moderate altitude may be more efficacious in improving parameters of dyslipidemia and circulating levels of adipokines than training at low altitude. Greater reductions of triglyceride and leptin levels in moderate altitude may suggest that metabolic responses to hypobaric and hypoxic conditions may translate into better short-term improvement of dyslipidemia and thus of MetS. Based on these preliminary observations, the efficacy and safety of hiking vacations at moderate altitude for improving diseases parameters in patients with MetS should to be further investigated in larger scale interventional trials.
